# Supplementary figures and images for: Endemism and diversity of small mammals along two neighboring Bornean mountains
Source: PeerJ. 2019 Oct 8;7:e7858. doi: 10.7717/peerj.7858 (PMC6788440; doi:10.7717/peerj.7858)

**Mt. Kinabalu**

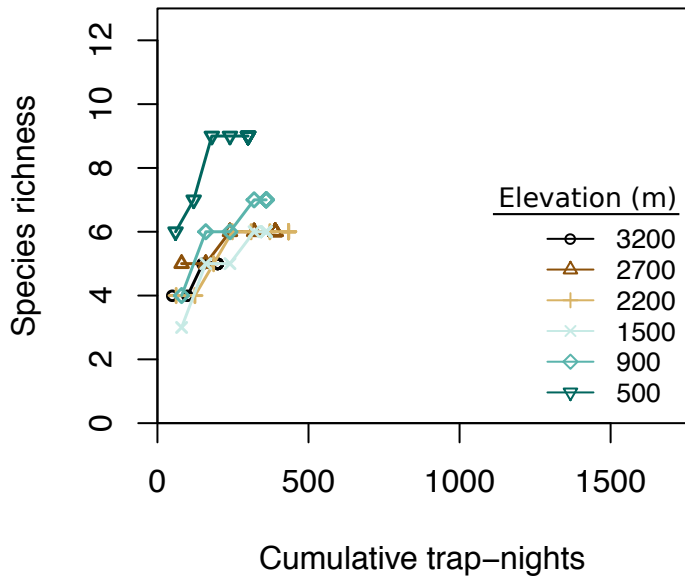

**Mt. Tambuyukon**

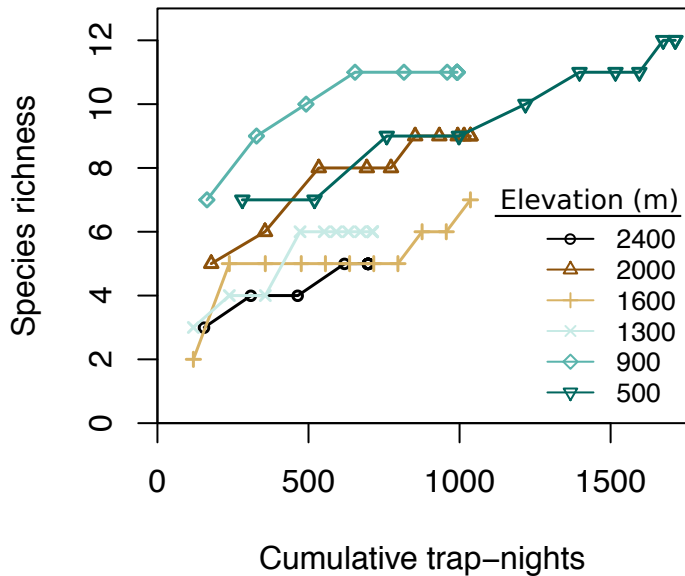

Supplement: Supplemental Information 2 [file peerj-07-7858-s002.pdf]

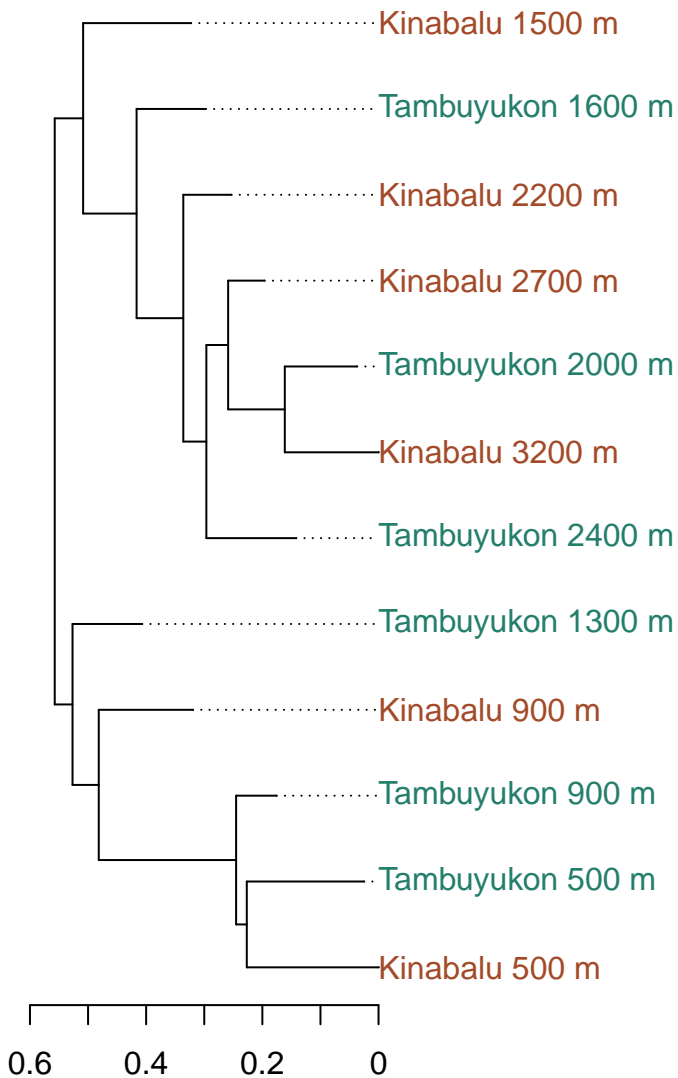

Supplement: Supplemental Information 4 [file peerj-07-7858-s004.pdf]
