# Supplementary material for: Endemism and diversity of small mammals along two neighboring Bornean mountains
Source: PeerJ. 2019 Oct 8;7:e7858. doi: 10.7717/peerj.7858 (PMC6788440; doi:10.7717/peerj.7858)
Supplement: Supplemental Information 3 [file peerj-07-7858-s003.pdf]

Figure S2: Photographs from camera traps.

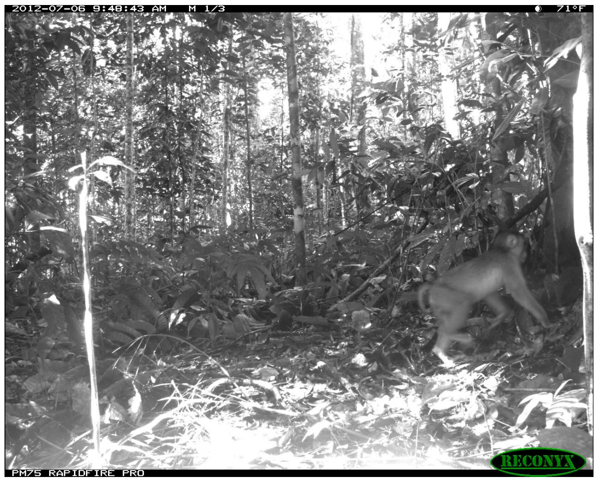

S2.1. Camera 1: *Macaca nemestrina*

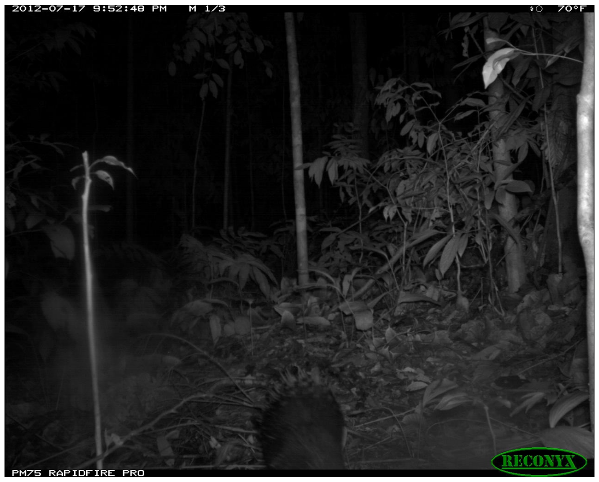

S2.2. Camera 1: *Hystrix brachyura*

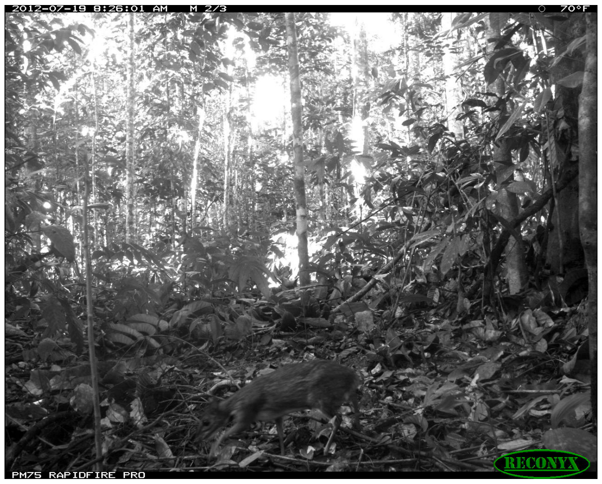

S2.3. Camera 1: *Tragulus* sp.

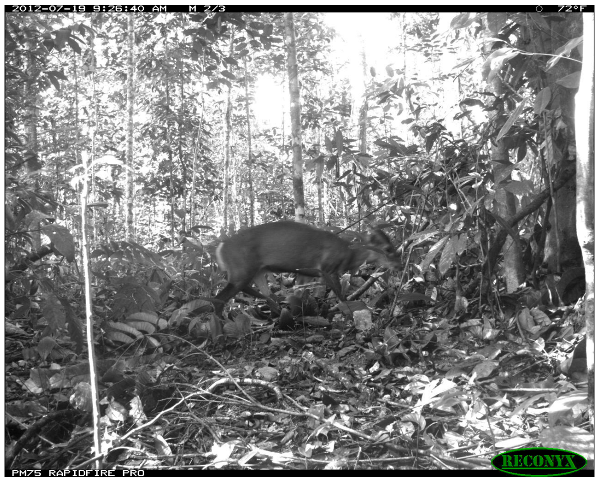

S2.4. Camera 1: *Muntiacus* sp.

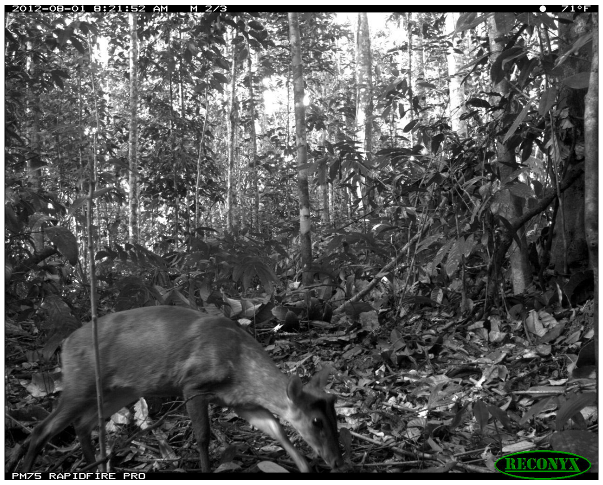

S2.5. Camera 1: *Rusa unicolor*

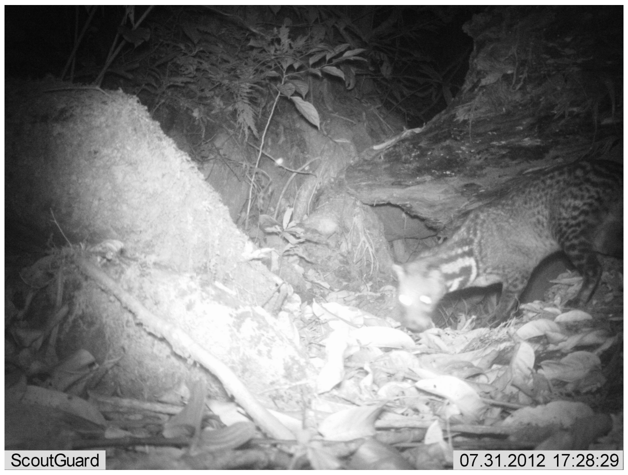

S2.6. Camera 2: *Viverra tangalunga*

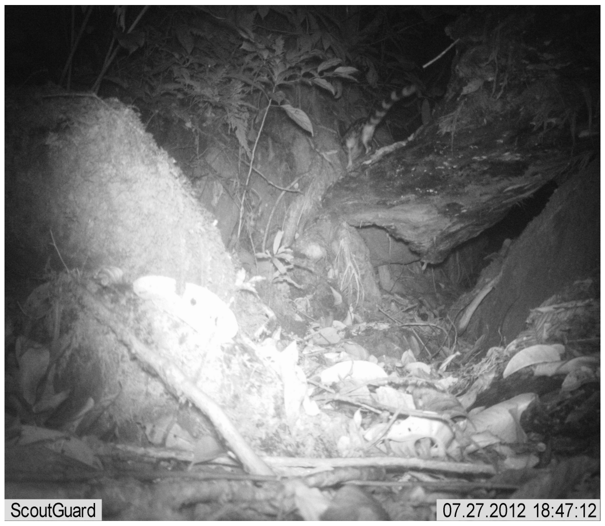

S2.7. Camera 2: *Prionodon lisang*

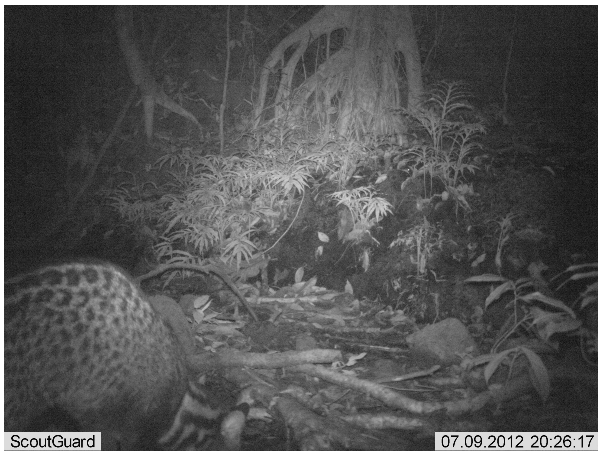

S2.8. Camera 3: *Viverra tangalunga*

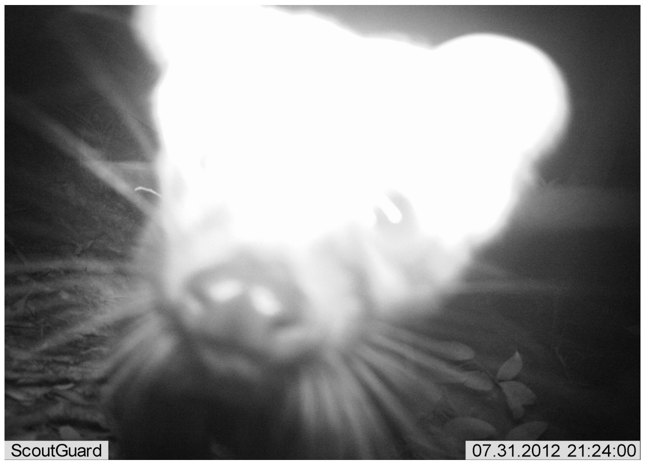

S2.9. Camera 4: *Viverra tangalunga*

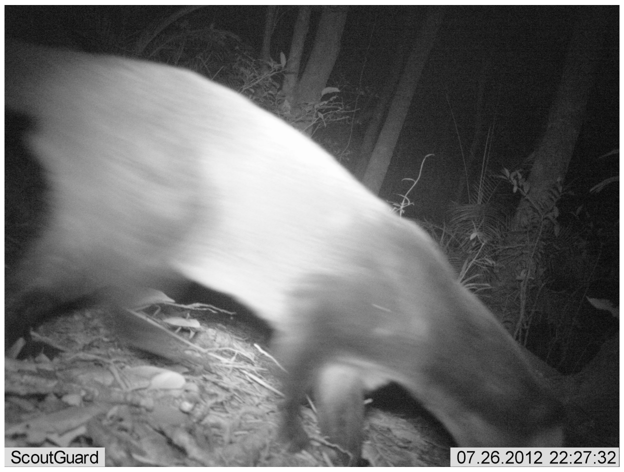

S2.10. Camera 4: *Paguma larvata*
