# Supplementary material for: Endemism and diversity of small mammals along two neighboring Bornean mountains
Source: PeerJ. 2019 Oct 8;7:e7858. doi: 10.7717/peerj.7858 (PMC6788440; doi:10.7717/peerj.7858)
Supplement: Supplemental Information 5 — Small mammals trapped during field surveys. Mt. Tambuyukon was surveyed at 500, 900, 1,300, 1,600, 2,000 and 2,400 m. Mt. Kinabalu was surveyed at 500, 900, 1,500, 2,200, 2,700, 3,200 m. Columns are headed with the elevation (m), where the same (or similar) elevation was sampled between the two mountains (Mt. Tambuyukon/Mt. Kinabalu). [file peerj-07-7858-s005.docx]

**Table S1: Summary table of species per elevation**

Small mammals trapped during field surveys. Mt. Tambuyukon was surveyed at 500, 900, 1,300, 1,600, 2,000 and 2,400 m. Mt. Kinabalu was surveyed at 500, 900, 1,500, 2,200, 2,700, 3,200 m. Columns are headed with the elevation (m), where the same (or similar) elevation was sampled between the two mountains (Mt. Tambuyukon/Mt. Kinabalu).

|  | 500 | 900 | 1,300 | 1,600  /1,500 | 2,000/ 2,200 | 2,400 | 2,700 | 3,200 | Total |
| --- | --- | --- | --- | --- | --- | --- | --- | --- | --- |
| *Callosciurus prevostii* | 0/2 | - | - | - | - | - | - | - | 2 |
| *Chiropodomys pusillus* | 1/0 | - | - | - | - | - | - | - | 1 |
| *Crocidura* sp. | - | - | - | 1/0 | - | - | - | - | 1 |
| *Hylomys suillus* | - | - | - | - | 3/2 | - | 3 | 3 | 11 |
| *Lenothrix canus* | 1/0 | - | - | - | - | - | - | - | 1 |
| *Leopoldamys sabanus* | 7/0 | 1/3 | 4 | 1/5 | 0/2 | - | 1 | - | 24 |
| *Maxomys alticola* | - | - | - | 1/0 | 12/3 | 11 | 5 | - | 32 |
| *Maxomys ochraceiventer* | 0/1 | 4/5 | - | - | 3/0 | - | - | - | 13 |
| *Maxomys rajah* | 19/1 | 2/1 | 2 | - | - | - | - | - | 25 |
| *Maxomys surifer* | 1/1 | 2/1 | - | - | - | - | - | - | 5 |
| *Maxomys whiteheadi* | 15/5 | 4/0 | 4 | 3/3 | 2/0 | - | - | - | 36 |
| *Melogale everetti* | - | - | - | - | 1/0 | - | - | 1 | 2 |
| *Niviventer cremoriventer* | 4/13 | 1/0 | 1 | - | - | - | - | - | 19 |
| *Niviventer rapit* | - | - | - | 1/0 | 0/1 | 1 | - | - | 3 |
| *Rattus baluensis* | - | - | - | - | 2/0 | 20 | 15 | 11 | 48 |
| *Rattus exulans* | 1/0 | - | - | - | - | - | - | - | 1 |
| *Rattus tanezumi* | - | - | - | 0/1 | 0 | 0 | 0 | 0 | 1 |
| *Suncus sp.* | 1/0 | - | - | - | - | - | - | - | 1 |
| *Sundamys infraluteus* | - | - | - | 0/4 | 1/0 | - | - | - | 5 |
| *Sundamys muelleri* | 23/7 | 1/0 | - | - | - | - | - | - | 31 |
| *Sundasciurus everetti* | - | 0/1 | 11 | 2/2 | 7/7 | 4 | 11 | 7 | 52 |
| *Sundasciurus jentinki* | - | 0/1 | - | - | - | - | - | - | 1 |
| *Sundasciurus lowii* | - | 1/0 | - | - | - | - | - | - | 1 |
| *Tupaia longipes* | 2/1 | 1/0 | - | - | - | - | - | - | 4 |
| *Tupaia minor* | 0/2 | - | - | - | - | - | - | - | 2 |
| *Tupaia montana* | - | 4/8 | 31 | 13/21 | 24/20 | 31 | 25 | 7 | 183 |
| *Tupaia tana* | 3/0 | 3/0 | - | - | - | - | - | - | 6 |
| Number of species: | 15 | 13 | 6 | 9 | 11 | 5 | 6 | 5 | 26 |
