# Supplementary material for: Endemism and diversity of small mammals along two neighboring Bornean mountains
Source: PeerJ. 2019 Oct 8;7:e7858. doi: 10.7717/peerj.7858 (PMC6788440; doi:10.7717/peerj.7858)
Supplement: Supplemental Information 6 — Diversity calculations for both mountains, across elevations (H′, Shannon diversity index; D, Simpson diversity index; S, species richness; J′, Pielou’s evenness index). [file peerj-07-7858-s006.docx]

**Table S2:**

Diversity calculations for both mountains, across elevations (H’, Shannon diversity index; D, Simpson diversity index; S, species richness; J’, Pielou’s evenness index).

|  | Elevation (m) | H’ | D | S | J’ |
| --- | --- | --- | --- | --- | --- |
| Mt. Kinabalu | 500 | 1.75 | 0.24 | 9 | 0.79 |
|  | 900 | 1.60 | 0.26 | 7 | 0.82 |
|  | 1,500 | 1.30 | 0.38 | 6 | 0.73 |
|  | 2,200 | 1.28 | 0.38 | 6 | 0.71 |
|  | 2,700 | 1.45 | 0.28 | 6 | 0.81 |
|  | 3,200 | 1.40 | 0.27 | 5 | 0.87 |
| Mt. Tambuyukon | 500 | 1.89 | 0.20 | 12 | 0.76 |
|  | 900 | 2.27 | 0.11 | 11 | 0.95 |
|  | 1,300 | 1.23 | 0.40 | 6 | 0.69 |
|  | 1,600 | 1.36 | 0.38 | 7 | 0.70 |
|  | 2,000 | 1.66 | 0.26 | 9 | 0.76 |
|  | 2,400 | 1.24 | 0.33 | 5 | 0.77 |
