# Supplementary material for: Endemism and diversity of small mammals along two neighboring Bornean mountains
Source: PeerJ. 2019 Oct 8;7:e7858. doi: 10.7717/peerj.7858 (PMC6788440; doi:10.7717/peerj.7858)
Supplement: Supplemental Information 7 — Pairwise dissimilarity between trapping locations based on Sorensen index (βsor), and its decomposition into the turnover (βsim) and nestedness (β sne) components. [file peerj-07-7858-s007.rtf]

$Kinabalu$beta.sim      500  900 1500 2200 2700900  0.57                    1500 0.83 0.50               2200 1.00 0.50 0.50          2700 1.00 0.50 0.50 0.17     3200 1.00 0.60 0.60 0.40 0.20$beta.sne      500  900 1500 2200 2700900  0.05                    1500 0.03 0.04               2200 0.00 0.04 0.00          2700 0.00 0.04 0.00 0.00     3200 0.00 0.07 0.04 0.05 0.07$beta.sor      500  900 1500 2200 2700900  0.62                    1500 0.87 0.54               2200 1.00 0.54 0.50          2700 1.00 0.54 0.50 0.17     3200 1.00 0.67 0.64 0.45 0.27$Tambuyukon$beta.sim      500  900 1300 1600 2000900  0.27                    1300 0.33 0.17               1600 0.71 0.57 0.33          2000 0.89 0.67 0.50 0.43     2400 1.00 0.80 0.60 0.20 0.20$beta.sne      500  900 1300 1600 2000900  0.03                    1300 0.22 0.25               1600 0.08 0.10 0.05          2000 0.02 0.03 0.10 0.07     2400 0.00 0.08 0.04 0.13 0.23$beta.sor      500  900 1300 1600 2000900  0.30                    1300 0.56 0.41               1600 0.79 0.67 0.38          2000 0.90 0.70 0.60 0.50     2400 1.00 0.88 0.64 0.33 0.43
